# Extraction and Conversion of Carboxymethyl Cellulose from Okara Soybean Residue via Soda AQ Pulping: Integration of Predictive Models and Process Control

**DOI:** 10.3390/polym17060777

**Published:** 2025-03-14

**Authors:** Preeyanuch Srichola, Titinunt Kitrungrotsakul, Kuntawit Witthayolankowit, Chaiyaporn Sampoompuang, Keowpetch Lobyaem, Prapakorn Khamphakun, Rawiwan Tumthong

**Affiliations:** 1 Cellulose for Future Materials and Technologies Special Research Unit, Department of Biotechnology, Faculty of Agro-Industry, Kasetsart University, Chatuchak, Bangkok 10900, Thailand; 2 Kasetsart Agricultural and Agro-Industrial Product Improvement Institute, Kasetsart University, Chatuchak, Bangkok 10900, Thailand; 3 Research Center for Space Computing System, Zhejiang Lab, Hangzhou 311111, China; 4 Department of Chemistry, Faculty of Science, Kasetsart University, Chatuchak, Bangkok 10900, Thailand

**Keywords:** okara, soda AQ, pulp, random forest, gradient boosting

## Abstract

This study investigates the effect of bases NaOH and KOH on okara, the soybean residue, in conventional pulping, based on 136 pulping conditions used as a dataset for random forest regression and gradient boosting predictive models. Okara CMC was formed and identified using Fourier-transform infrared spectroscopy (FTIR) to demonstrate a wide range of applications comparable to commercial CMC, with a low degree of substitution. The quality of okara pulp after basic pulping was analyzed based on the extracted cellulose yield and remaining protein content. The optimized pulping condition was a mixture of NaOH and KOH at a 30% concentration, resulting in an extracted cellulose yield of 24.5 wt% and a remaining protein content of 25.1%. The obtained okara pulp was converted into okara CMC with a controllable degree of substitution. The implemented dataset was used to train two predictive models: random forest regression and gradient boosting, to forecast key parameters for pulping (NaOH, KOH, AQ, and H_2_O). Both models demonstrated excellent prediction performance, with R^2^ values of 0.94 and 0.89, respectively, and showed similar residuals and predicted values. The close clustering of residuals around zero, along with the sharp and narrow curves observed, indicates that both the random forest and gradient boosting models provide precise and reliable predictions. The localized deviations observed in the residuals suggest that these models effectively capture detailed patterns in the data, leading to minimized prediction errors within specific ranges.

## 1. Introduction

Global soybean production for the 2023–2024 season is projected to exceed 380 million metric tons, with the primary production areas located in the Americas and Asia. This forecast is subject to change based on factors such as climate, planting area, and global trade dynamics. To meet the growing demand, soybean production is expanding, which in turn impacts the climate, water usage, resource consumption, and the environment [1]. One of the major concerns is the accumulation of soybean waste, which has become a significant contributor to environmental issues. Rather than focusing solely on soybean sustainability, the rapid growth of the soybean industry, driven by the global demand for food, has led to a significant increase in the consumption of food alternatives made from soybeans [2]. During the production of soymilk and tofu, a byproduct known as okara soybean residue is generated [3]. For every ton of tofu produced, approximately more than 1 ton of okara is generated, resulting in over 3,000,000 tons of okara annually in Asia [4,5,6,7]. However, okara soybean residue still contains approximately 50% fiber, 10% fat, and 25% protein. It can be utilized in animal feed and even as a fertilizer in agriculture [8,9,10]. Using okara for pulp production to create cellulose or fiber offers a promising approach to reducing waste, lowering costs, and creating sustainable products. As technology and methods continue to improve, the efficiency and quality of okara-based pulp products are likely to increase, broadening their potential applications. By utilizing okara as a substrate for pulp, economic benefits can be gained by transforming a waste product into a valuable resource. Rather than incurring costs for waste disposal, companies can generate additional revenue streams by producing value-added products [11]. This initiative focuses on promoting sustainability and efficiency across the entire soybean value chain. In response to environmental issues, significant research has been conducted on recycling okara, a byproduct of soybean processing, into cellulose pulp [12]. Chemical pulping is a process used to separate cellulose pulp from wood or other plant materials (non-wood), primarily for the production of pulp, paper, and other cellulose-based products. The most common types of chemical pulping processes are kraft, sulfite, soda, and soda AQ. These processes involve the use of chemicals such as sodium hydroxide (NaOH), potassium hydroxide (KOH), sodium sulfide (Na_2_S), sulfur dioxide (SO_2_), and anthraquinone (AQ) in the cooking liquor. The soda AQ pulping process is a chemical method used for non-wood pulp, with NaOH as the cooking chemical. NaOH breaks down lignin, which binds cellulose fibers together in non-wood materials. The concentration of NaOH affects the viscosity and flow characteristics of the pulp slurry, and effective lignin removal leads to a higher yield of pure cellulose, which is crucial for producing high-quality paper. Moreover, proper NaOH levels ensure optimal pulp consistency, facilitating smoother processing and sheet formation. Hence, managing NaOH concentration and its mixing with H_2_O is crucial for balancing the desired paper properties with process efficiency. Careful control of NaOH levels ensures that the pulping process achieves the necessary quality and performance standards while maintaining operational efficiency. The addition of AQ in the soda AQ process helps decrease carbohydrate degradation. The use of AQ can reduce the amount of soda or other chemicals needed, leading to more efficient processing and improving the overall quality of the pulp. This versatility makes it a valuable method in the paper and pulp industry [13,14,15,16]. Moreover, KOH is also used as a primary alkaline agent to effectively break down lignin and separate cellulose fibers during the pulping process. KOH pulping can be more environmentally friendly compared to some other chemical processes, depending on how the process is managed and the disposal of byproducts. The resulting pulp typically has favorable properties for producing high-strength and high-quality paper. This method improves the quality and efficiency of pulp production, resulting in high-quality paper with good strength properties [17]. These chemicals are expensive and play a vital role in breaking down lignin and separating cellulose fibers. Proper washing helps recover these chemicals from the spent liquor, allowing them to be reused in the pulping process, which reduces costs and minimizes environmental impact. During the pulping process, various impurities such as lignin, extractives, and other organic and inorganic substances are dissolved or suspended in the cooking liquor. If not effectively washed away, these impurities can negatively impact pulp quality, cause equipment corrosion, and result in increased chemical usage downstream [18,19,20,21]. Separating cellulose pulp from okara, a byproduct of soybean processing, presents significant challenges due to the complex nature of these materials. Okara has a high protein content, ca. 25%, and a high content of natural cellulose, which is the major component at ca. 50%. The distinct chemical compositions and physical properties of these components contribute to the difficulty in finding a single solvent or method that can selectively dissolve one component while leaving the other intact. This complexity makes achieving a clean and efficient separation process challenging. Researchers have been exploring various techniques to overcome these difficulties and develop effective methods for isolating cellulose fibers from okara soybean residue. This article will also highlight the extraction and conversion of carboxymethyl cellulose (CMC) from okara soybean residue using the pulping process (soda AQ) and modification methods, showcasing it as an example of modified cellulose [22].

However, few studies have been carried out on the effect of the chemical processes used to make cellulose pulp. The chemical processes used do not account for the chemical demand for wood and non-wood raw materials for making cellulose pulp. Therefore, integrating predictive models and process control systems into pulping operations is essential for achieving operational excellence, maximizing productivity, ensuring product quality, and meeting sustainability goals [23]. Conducting comprehensive assessments to determine the chemical demand for wood and non-wood raw materials under varying process conditions is one of the best ways to improve the efficiency of the pulping process [24]. Evaluating the environmental impact of pulping processes in relation to the chemical demand of raw materials is equally important [25].

The Institute for Research and Development of Agricultural Products and Agroindustry has long focused on the production of biomass and biodegradable materials [26,27,28]. Building on this knowledge, the purpose of this study was to examine the chemical separation of cellulose and CMC modification from okara soybean residues. Additionally, the research aimed to fill a gap by exploring the relationship between predictive models and process control in the soda AQ pulping of okara. The study emphasizes how predictive modeling can enhance process efficiency, product quality, and resource utilization during pulping. Using a specific pulping process as a case study, the article provides practical insights into applying predictive modeling in real-world scenarios, focusing on optimizing temperature control, chemical dosing, and pulp quality monitoring. The findings aim to deepen the understanding of how predictive modeling can improve the sustainability and efficiency of okara soybean residue pulping. Ultimately, the goal is to offer actionable recommendations for pulp mills and industry stakeholders seeking to enhance their operations with predictive analytics and process control strategies. This not only helps reduce the amount of okara waste, but also minimizes processes that contribute to environmental harm.

## 2. Materials and Methods

### 2.1. Soda AQ Pulping Process Description

**Figure 1:**
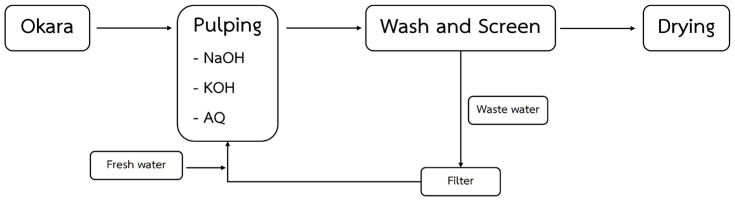
Soda AQ pulping process of okara soybean residue.

The general process for soda AQ pulping is shown in Figure 1. During the pulping process, okara soybean residue was fixed as the limiting agent, and the equivalent amount of base (NaOH and KOH) was varied in each pulping condition, starting from 0 to 50 wt% of okara residue (see Supplementary Materials). In some conditions, a small amount of AQ (0–5 wt% of okara residue) was added as a catalyst for pulping (see Supplementary Materials). The mixture was pulped at 85 °C for 1–4 h using water (H_2_O) as a solvent to yield pulp slurry across a total of 136 pulping conditions. The obtained okara pulps were filtered through a screener to remove large particles and impurities and then pressed into sheets to remove excess water, improving their density and texture. The pressed sheets were dried for further carboxymethyl modification. To utilize the wastewater generated during the pulping process, wastewater from drainage was filtered and partially reused for pulping to reduce the environmental impact and increase resource efficiency.

### 2.2. Preparation of Carboxymethyl Cellulose from Okara Soybean Pulp

**Figure 2:**
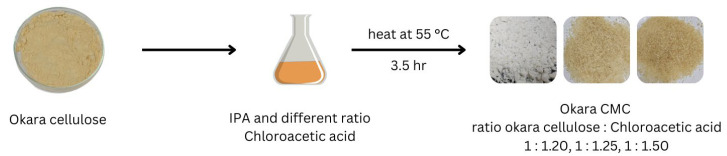
Production of carboxymethyl cellulose from okara soybean residue.

The general process for the carboxymethyl modification of okara soybean pulp is shown in Figure 2. The extracted okara pulp was dissolved in isopropyl alcohol (IPA), and the protecting reagent, chloroacetic acid, was added in 1.20, 1.25, and 1.50 equivalents of okara pulp, respectively. These three mixtures were heated at 55 °C for 3.5 h to complete the alcohol modification, resulting in carboxymethyl cellulose (CMC) from okara. The obtained CMC was washed with ethanol and dried at room temperature overnight for further analysis.

### 2.3. Preparation of CMC Film from Okara

**Figure 3:**
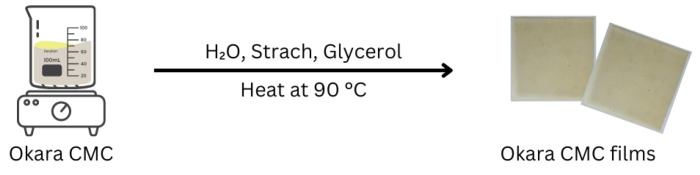
Preparation of CMC film from okara.

Okara-based CMC films were prepared based on the modified method of Sukyai et al. (2018) [29]. The general method started with a mixture of CMC (3 g) and modified starch (3 g) in a 1:1 ratio; 100 mL of H_2_O were then stirred in, and the mixture was heated at 60 °C for 10 min. After the solution became homogeneous, different amounts of glycerol (0, 0.5, 1 mL) were added to the reaction and stirred for 5 min. The mixtures were then poured into a glass container and dried in a hot air oven to form the films, as shown in Figure 3.

### 2.4. Characterizations

The prepared okara-based CMC films were characterized as follows:

Functional groups of CMC films: Fourier-transform infrared (FT-IR) spectroscopy (Nicolet IR200 FT-IR Infrared Spectrometer, Thermo Scientific, Waltham, MA, USA) was used in the study to analyze infrared radiation absorbance. The measurement mode used was absorption spectroscopy, with interval scanning data set to an intensity of 0.02 and a measurement range of 450–4000 cm^−1^. The materials were placed in a sample cell, and the spectra were recorded.

(1) $$A=log\frac{I_{0}}{I}=\varepsilon bc,$$

where 

*A* = absorbance;

*ε* = molar absorptivity unit dm^3^ cm^−1^ g^−1^;

*c* = concentration unit g dm^−3^ or mol L^−1^ or molar;

*I*_0_ = light intensity;

*I* = light intensity when passing the sample;

*b* = thickness of the sample;

Degree of substitution of carboxymethyl cellulose: The DS determination of okara CMC was modified from Kono et al., 2018 [30]. CMC (30 mg) was acidified with a 1:1 ratio of ethanol and hydrochloric acid (4 mL). The solid CMC was filtered and rinsed with water and ethanol. Then, the CMC was basified with 0.1 M NaOH (1 mL) and titrated with 0.1 M HCl using phenolphthalein as an indicator. The CM content (%) and DS were calculated using the following equations (Equations (2) and (3)):

CM content (%) = [(V_0_ − V_n_) × *M* × 0.059 × 100]/*m*, (2)

where V_0_ is the amount of HCl used to titrate the blank solution, V_n_ is the amount of HCl used to titrate the samples, *M* is the molar concentration of HCl, and *m* is the sample amount;

DS = (162 × CM content (%))/[5900 − (58 × CM content (%))],(3)

where the value of 162 g mol^−1^ is the molar mass of the cellulose AGU and 58 g mol^−1^ is the molar mass increase for the CM group substitution per hydroxyl group.

### 2.5. Case Study: Process Variables

This study also focused on implementing and training two predictive models, random forest regression and gradient boosting models, to forecast key parameters (NaOH, KOH, AQ, and H_2_O) and examine the effect of the chemical processes used to make wood and non-wood pulp [31,32]. Collecting data on key process variables, such as chemical concentrations and okara feedstock properties, is a crucial step in understanding and optimizing the pulping process.

- NaOH—the concentration of NaOH used in the pulping process. NaOH is commonly used as a cooking liquor to break down fat, protein, and lignin in the okara feedstock.
- KOH—the concentration of KOH used, if applicable. KOH is used in addition to or instead of NaOH for pulping.
- AQ—the concentration of AQ, which serves as a pulping additive to improve delignification efficiency.
- H_2_O—the amount of H_2_O used in the pulping process, which serves as a solvent for the chemicals and facilitates the pulping reaction.

### 2.6. Data Collection, Model Development, and Processing

To gather data on the pulping process, key process variables such as the chemical concentrations of NaOH, KOH, AQ, and H_2_O, along with properties of the okara feedstock, were collected, as shown in Table 1. In this article, the chemical data used were obtained from records collected from the pulping section, comprising data from over 136 conditions. These samples likely included measurements of the various chemicals used in the soda AQ pulping process of okara, such as alkali (soda) and anthraquinone (AQ), among others. The large dataset collected from these samples served as the basis for analyzing the relationship between chemical variables and process outcomes. By leveraging predictive modeling techniques, researchers could extract valuable insights from these data to optimize chemical dosing, identify potential process inefficiencies, and improve overall pulp quality. The utilization of such a comprehensive dataset underscores the importance of data-driven approaches in modern pulp manufacturing.

### 2.7. Machine Learning Implementation

Due to the limitations of our dataset, a model to predict the yield rate from NaOH, KOH, AQ, and H_2_O using random forest [31] and gradient boosting [32] was trained. Before training, the input and output variables were normalized to enclose the input data in a range of [0, 1]. In the implementation of the random forest regression model, key parameters were meticulously configured to enhance predictive performance and ensure model robustness, as shown in Table 2. The number of estimators was set to 100, balancing the trade-off between accuracy and computational efficiency. To prevent overfitting, the maximum depth of each tree was constrained to 8. Additionally, a minimum of 5 samples per split and 2 samples per leaf were specified, ensuring that splits and leaves were formed based on sufficient data. The “auto” setting for the maximum number of features considered at each split was employed, allowing the model to utilize all available features for optimal split decisions. Bootstrap sampling was enabled, promoting model diversity by training each tree on a different subset of the dataset. The mean squared error (MSE) criterion was utilized to evaluate the quality of splits, which is particularly well-suited for regression tasks. This metric measures the average squared difference between the actual outputs and the predicted outputs. These parameter settings collectively aimed to optimize the model’s performance while maintaining generalization capability. To evaluate the performance of the random forest and gradient boosting models, the R^2^ score was used for evaluation.

The R^2^ value was calculated according to the following equation:

$$R^{2}=1-\frac{\sum_{i=1}^{n}{\left(y_{i}-{\hat{y}}_{i}\right)}^{2}}{\sum_{i=1}^{n}{\left(y_{i}-{\bar{y}}_{i}\right)}^{2}}$$

where *n* represents the number of datapoints, *y* is the actual output of data, *i* is the element, *ŷ* is the predicted output from the model, and *ȳ* is the mean of the actual output. The random forest, gradient boosting, and evaluation metrics reported in this paper were implemented in the Python 3.9 environment using the scikit-learn library.

## 3. Results and Discussion

Okara soybean residue was pretreated by grinding, followed by soda AQ pulping under various conditions based on the weight percentages of NaOH, KOH, AQ, and H_2_O. The hydrolysis of okara under basic conditions was successfully carried out in a total of 136 conditions (see Supplementary Materials), and the primary data from pulping were used for machine learning to optimize the concentration and effect of chemicals during the pulping process. During hydrolysis, basic conditions with a mixture of NaOH and KOH were used as nucleophiles to cleave peptide bonds and ester bonds in okara, extracting the insoluble cellulose fibers intact [33]. In the presence of AQ as a catalyst, it is well-known that NaOH is the common base for biomass valorization, and it has been used in the pulping industry for more than a decade [34]. To achieve a sustainable process and produce high-quality cellulose for various applications, there is debate about using more environmentally friendly bases, such as KOH [35,36,37]. Since both NaOH and KOH are strong bases, the mixture of these bases catalyzed the hydrolysis of okara to compare the effect of extracting cellulose from okara and maximizing the yield of okara pulp. Some selected pulping conditions are shown in Table 3. The pulping temperature was fixed at 85 °C, and all pulping was carried out for 1 h. For the control condition, okara was pulped in water without any base, and the yield of cellulose was 78.8 wt%, representing the hydrolysis of water-soluble cellulose, such as hemicellulose [38,39]. The remaining protein content in the okara pulp samples was analyzed to determine the most effective way to separate the protein and fiber components of the okara. The pulping with a high concentration (50%) of NaOH (Table 3, entry 1) yielded 26.5 wt% of cellulose, which was set as a benchmark for the pulping condition due to the high concentration of NaOH being excessive for peptide and ester hydrolysis. To improve the sustainability and efficiency of the pulping conditions, KOH was mixed with NaOH in different ratios, and AQ was also added to optimize cellulose yield. However, the results showed that pulping okara with 50% KOH in water yielded 34.5 wt% of cellulose, representing the poor hydrolysis ability of KOH toward peptide bonds and resulting in the highest remaining protein content at 37.1%. When NaOH was mixed with KOH for okara pulping, the efficiency of hydrolysis was slightly improved, and the total concentration of base in the pulping solution could be reduced. At a 30% base ratio of 2:1 NaOH and KOH, the pulping yielded 24.5 wt% of cellulose (Table 3, entry 5). To achieve the maximum hydrolysis of peptide and ester bonds in okara, the optimized pulping condition was 30% NaOH solution with 0.1% AQ as a catalyst, and the cellulose yield was 23.0 wt% (Table 3, entry 6). The quality of cellulose from 30% NaOH only and a 30% mixture of NaOH and KOH was comparable, with the remaining peptide bond content at 23.7% and 25.1%, respectively. Thus, adding KOH to improve the sustainability of pulping could be possible because the yield and quality of okara-pulped cellulose were comparable to the conventional pulping method with only NaOH. To study the effect of each chemical during the process, the 136 okara pulping conditions were used as input for model training to build predictive models using random forest regression and gradient boosting to forecast key parameters. To close the loop for the pulping process, wastewater generated during each pulping process was reused by filtration, and the pH of the water was measured for quality control.

To demonstrate the application of extracted okara cellulose after pulping, okara pulp from the mixture of NaOH and KOH pulping processes was converted into carboxymethyl cellulose via chloroacetic acid modification of the hydroxyl moiety on each monosugar unit. The modification of the hydroxyl group on okara pulp was optimized with different ratios of 1:20, 1:25, and 1:50 cellulose to chloroacetic acid, respectively, using isopropyl alcohol as the solvent (Table 4).

**Figure 4:**
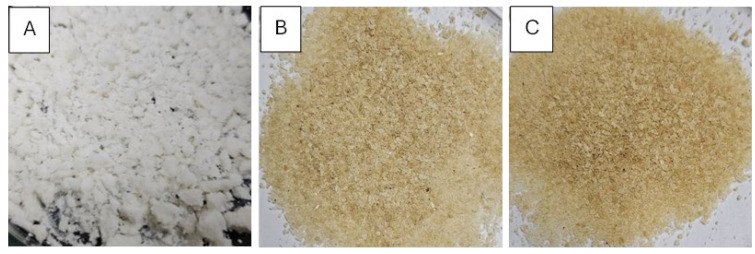
Okara CMC from the different modification conditions of Table 4. (**A**) Okara cellulose: chloroacetic acid, 1:1.20, entry A. (**B**) Okara cellulose: chloroacetic acid, 1:1.25, entry B. (**C**) Okara cellulose: chloroacetic acid, 1:1.50, entry C.

**Figure 5:**
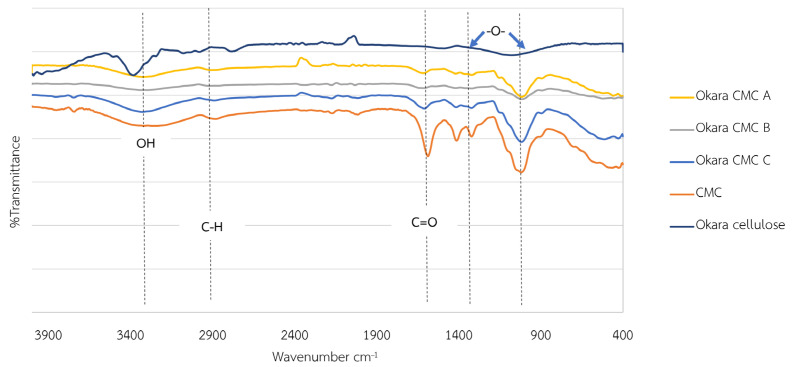
Fourier-transform infrared spectroscopy (FTIR) of carboxymethyl cellulose (CMC) from okara (okara pulp: chloroacetic acid, 1:1.20 (condition A), 1:1.25 (condition B), and 1:1.50 (condition C), respectively). CMC is commercial CMC.

**Figure 6:**
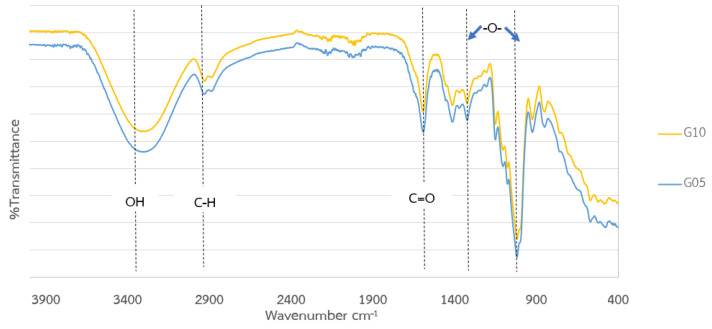
Fourier-transform infrared spectroscopy (FTIR) of the okara CMC film (G05: glycerol solution at volumes of 0.5 milliliters, G10: glycerol solution at volumes of 1 milliliter).

The yield of okara CMC from the different ratios of okara pulp and chloroacetic acid was almost the same at 23 wt%. However, the obvious difference between these okara CMC samples was the color, which was due to the difference in the degree of substitution (DS) of the carboxymethyl protecting group on each sample (Figure 4). With the low ratio of okara pulp to chloroacetic acid at 1:1.20, the okara CMC was white (similarly to normal cellulose) due to the low DS, showing that only a few hydroxyl groups were modified. When the ratio of okara pulp to chloroacetic acid was increased to 1:1.50, more hydroxyl groups were modified with the carboxymethyl group following Le Chatelier’s principle, which states that increasing the concentration of reactants shifts the reaction toward the products [40]. The DS of the obtained okara CMC could be controlled, with the range between 0.27 and 0.35. The DS of commercially available CMC is 0.32. Thus, okara CMC with optimized modification could be used in many applications that require a low substituted DS level, such as food adhesives and wound dressings [41,42,43,44]. The functional group analysis of the okara CMC using Fourier-transform infrared spectroscopy (FTIR) is shown in Figure 5. A comparison of the functional group spectra of the okara cellulose and okara CMC showed that the okara CMC also contained hydroxyl and hydrocarbon groups. However, okara CMC contained a carboxyl moiety (-COOH), which is evident in the peak of the carbonyl group, showing a higher intensity compared to the okara cellulose. When comparing the functional group spectra of carboxymethyl cellulose from okara in conditions A, B, and C, it was found that all the conditions exhibited similar functional groups—hydroxyl, carbonyl, and ether groups—but with varying intensities of the functional group peaks. The shift of the FTIR peak from 3500 to 3400 cm^−1^ represented the reduction of free hydroxyl groups from the okara cellulose, which could be free hydroxyl groups from water [45,46]. When comparing the functional group spectra of the okara CMC to commercial CMC, it was found that both spectra were similar, indicating that the okara CMC contained similar functional groups to commercial CMC. The process of preparing CMC film was modified based on the method by Sukyai et al. (2018) [29]. When the okara CMC was cast into films and dried at room temperature, the characteristics of the films were similar to commercial CMC, demonstrating the possibility of commercializing okara CMC as a CMC product for various applications. Additionally, the use of glycerol in the film formation process did not show a difference in the FTIR peaks of the okara CMC spectra (Figure 6).

**Figure 7:**
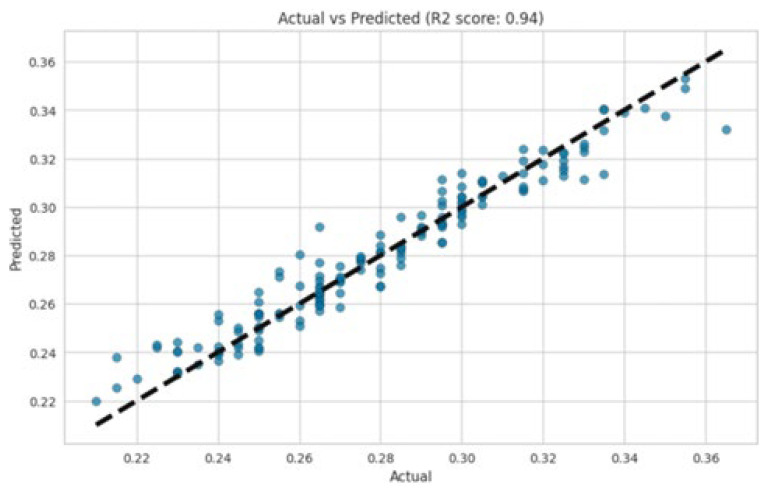
R^2^ value of the random forest.

**Figure 8:**
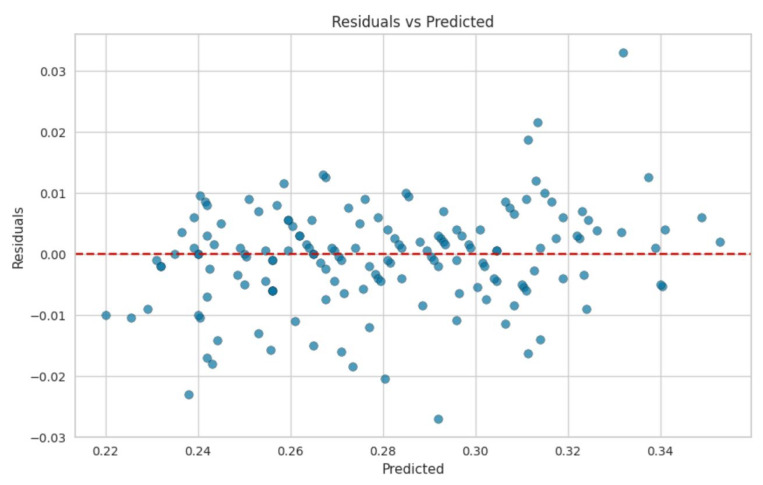
Residuals and predicted results of the random forest.

In this work, collecting detailed data on key process variables was essential for understanding and optimizing the pulping process. Specifically, variables such as NaOH, H_2_O, KOH, and AQ play crucial roles in determining the efficiency and effectiveness of the okara pulping process. Monitoring and adjusting the concentrations and conditions of these chemicals can help fine-tune the process, improving the quality and yield of the pulp. It was found that detailed data on these factors enable better control over the process and allow for more precise optimization of these datasets. The implementation of the random forest regression model presented a high R^2^ value, suggesting strong overall model performance. However, the presence of scattered results and high errors in specific instances indicates potential challenges related to dataset quality or model limitations. The R^2^ value, evaluated in Figure 7, was 0.94, indicating that high accuracy in modeling was achieved in the soda AQ pulping process (Figure 8). By carefully addressing data quality issues and exploring complementary modeling approaches, the reliability and robustness of the modeling efforts in the soda AQ pulping process for pulp and papermaking can be further enhanced.

**Figure 9:**
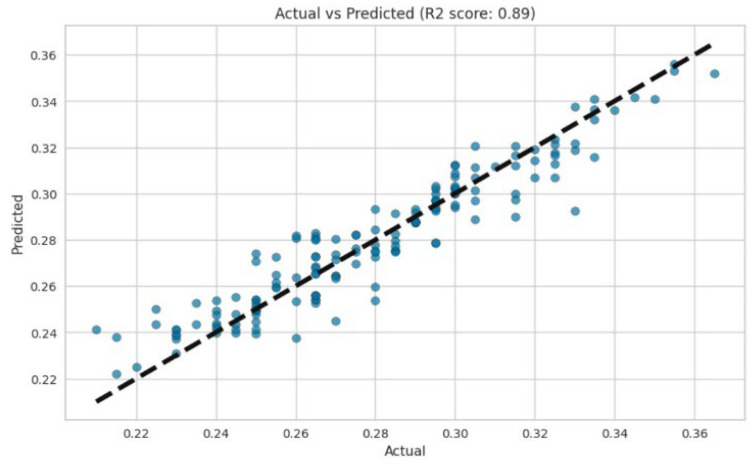
R^2^ value of gradient boosting.

**Figure 10:**
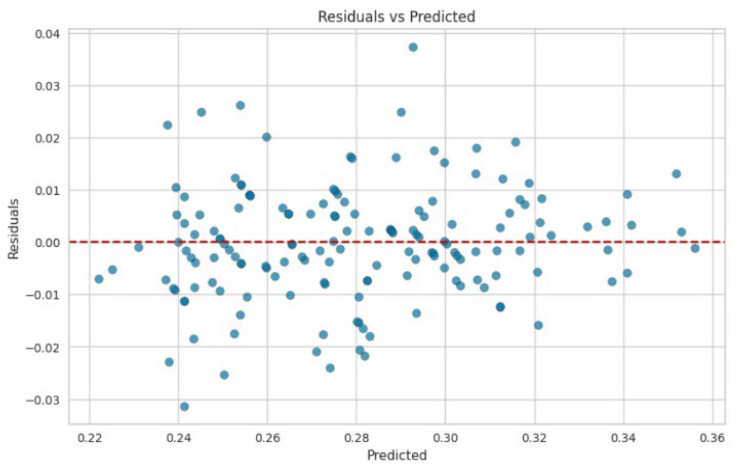
Residuals and predicted results of gradient boosting.

Gradient boosting is an algorithm that enhances model accuracy by iteratively minimizing errors through the use of negative gradients of the loss function. Its ability to achieve high R^2^ values is evident. Figure 9 and Figure 10 also show good results in terms of linear regression. An R^2^ value of 0.89 indicates that the gradient boosting model explained 89% of the variance in the target variable. This suggests that the model fitted the data well and captured a substantial amount of the variability present in the data. The random forest regression and gradient boosting models demonstrated higher accuracy (Figure 8 and Figure 10). The residuals and predicted results showed values within ±0.04, indicating that the errors for both models were within the second decimal place. These error values exceeded the control accuracy requirements for large-scale pulp production.

**Figure 11:**
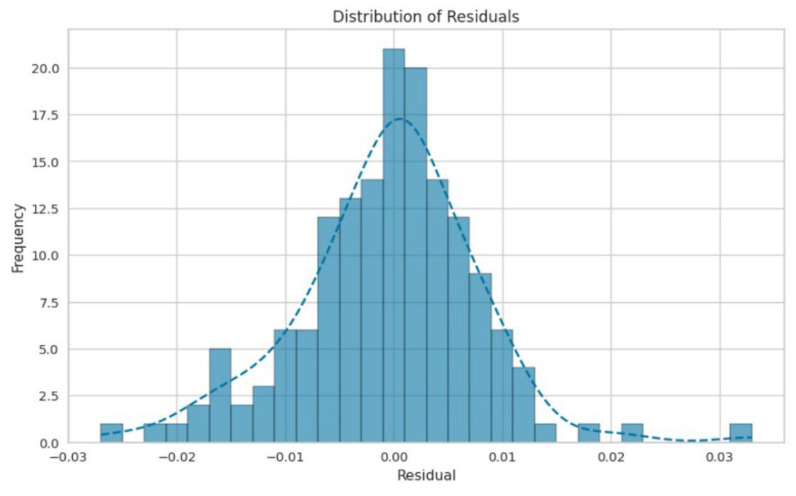
Distribution of residuals for the random forest.

**Figure 12:**
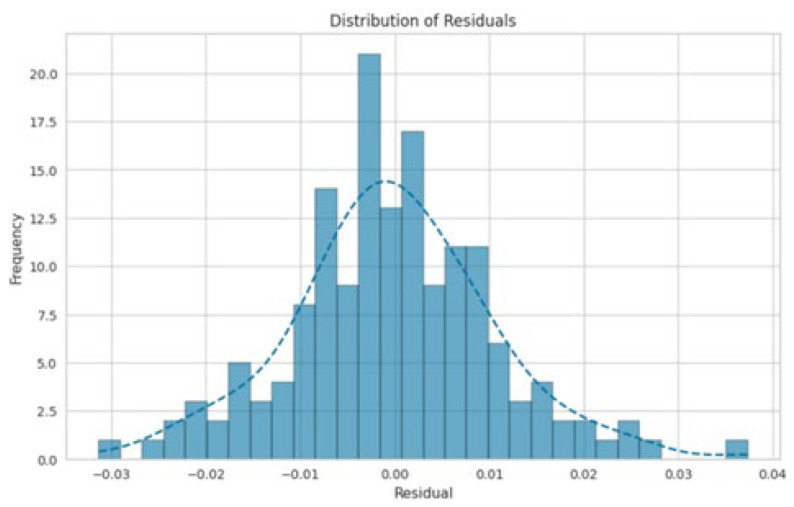
Distribution of residuals for gradient boosting.

Figure 11 and Figure 12 present an enlarged view of the distribution of residuals for the random forest and gradient boosting models, respectively. Both models exhibited residual distributions that were closely centered around zero and followed a bell-shaped normal curve. This indicates that the errors from these models were generally small and symmetrically distributed around zero. The frequency of residuals did not exceed 20, with most residuals falling within the range from −0.04 to 0.04. This narrow range of residuals suggests that both the random forest and gradient boosting models provided highly accurate predictions, with errors concentrated around zero. The impact of different chemicals on the pulping process, specifically focusing on NaOH mixed with H_2_O, appears to have a significant effect on the variables, which could be related to the quality or efficiency of the pulping process. Both the random forest and gradient boosting models showed that NaOH mixed with H_2_O had a high influence, as indicated by the “feature value.” This suggests that NaOH mixed with H_2_O was a crucial factor in this analysis.

**Figure 13:**
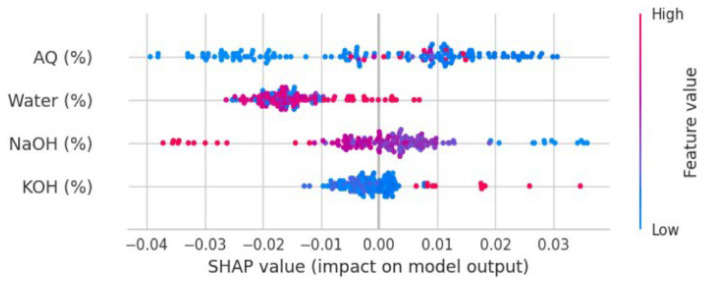
Features’ impact on the RF model output (SHAP value).

**Figure 14:**
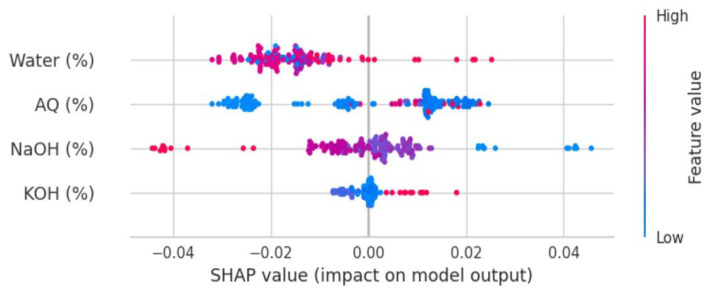
Features’ impact on Gradient Boosting model output (SHAP value).

Figure 13 and Figure 14 show a high feature value (depicted by the red dot), illustrating the impact of NaOH mixed with H_2_O graphically, reinforcing its importance in the models. Moreover, AQ and KOH were also studied. These chemicals were part of the analysis, and their effects on the pulping process were also evaluated. In the case of AQ and KOH, it was found that the feature value presented a relatively low value (depicted by the blue dot), indicating low influence in the pulping process. AQ and KOH represent feature importance or influence in these models, and the “relatively low value” indicates that AQ and KOH had less impact compared to NaOH mixed with H_2_O. The lower feature value for AQ and KOH suggested that these chemicals had a minor effect on the pulping process, as modeled by both the random forest and gradient boosting algorithms. In summary, NaOH mixed with H_2_O is a more influential variable in models for the pulping process compared to AQ and KOH, which have a smaller impact. This information can guide decisions in optimizing the pulping process or in understanding how different chemicals contribute to the final product.

When comparing the residuals of the linear regression model (depicted by the red line) with those of the random forest (depicted by the blue line) and gradient boosting models (depicted by the green line), it was observed that the residuals of the linear regression model exhibited a bell-shaped curve with large residual values ranging from 0 to 0.25. This pattern indicated that the linear regression model showed a lower reliability compared to the random forest and gradient boosting models. The large residuals and the bell-shaped distribution suggested that the linear regression model may not capture all the complexities of the data as effectively. This highlights the limitations of linear regression in this context and underscores the superior performance of the random forest and gradient boosting models, which likely provide more accurate and reliable predictions for the soda AQ pulping process.

In the residual analysis of the random forest and gradient boosting models, narrow, sharp curves were observed in the distribution. This pattern suggests that these models were effectively capturing specific patterns in the data. The residuals were clustered very close to zero, indicating that the models’ predictions were generally very accurate and close to the actual values for most instances.

**Figure 15:**
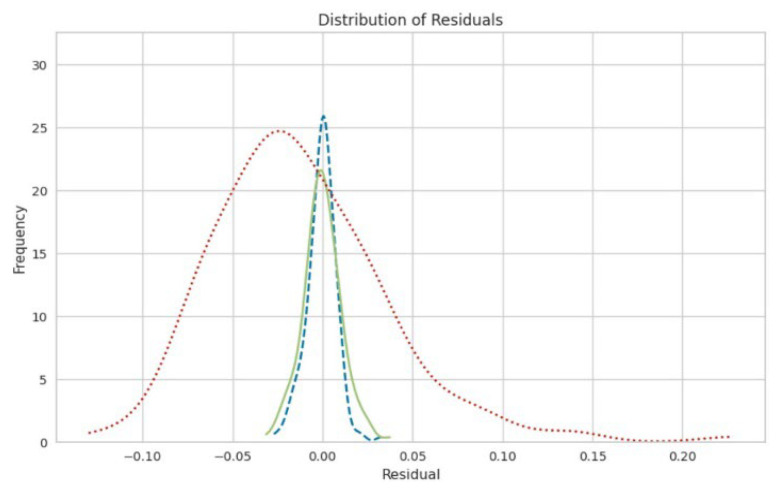
Comparison between the random forest (depicted by the blue line), gradient boosting (depicted by the green line), and linear regression models (depicted by the red line).

As illustrated in Figure 15, the residuals exhibited sharp, localized deviations within the range from 0 to 26. This implies that while the models perform well overall, they might be capturing particular patterns or anomalies within this limited range of values. The sharp curves suggest that these models fitted the data closely, revealing localized deviations that could reflect specific features or nuances in the dataset. Overall, the close clustering of residuals around zero and the sharp, localized deviations indicate that the random forest and gradient boosting models provided precise and reliable predictions, capturing detailed patterns and minimizing prediction errors within certain ranges.

The use of machine learning models, specifically the random forest and gradient boosting algorithms, proved valuable in quantifying the effects of different chemicals on the pulping process, providing actionable insights to improve product yield and quality. During the pulping process, a mixture of NaOH and KOH significantly performed peptide hydrolysis. Based on the data distribution in both models, NaOH was more important than KOH in terms of yield. Moreover, the amounts of AQ and water were also important for optimizing the pulping process. The results from both models precisely demonstrated the high deviation when the ratio of okara pulp to solvent was too high. Both models also showed the impact of AQ on the pulping process. The absence of an additional catalyst could lead to unpredictable pulping outcomes due to the limitations of lignin valorization during hydrolysis. By leveraging the features of both prediction models, they demonstrated more precise predictions for each chemical involved in the pulping process, providing more reliable conditions than conventional predictions made using linear regression. These models could serve as tools for optimizing okara pulping conditions to achieve high efficiency and sustainability in the industry.

## 4. Conclusions

This study successfully demonstrated the extraction of okara cellulose using a mixture of NaOH and KOH as a base for pulping without degrading the insoluble cellulose. The quality of okara pulp from both the conventional pulping process and the mixed base process was optimized, and the remaining protein in the okara pulp was not significantly different. The obtained okara cellulose was successfully converted into okara CMC, and the degree of substitution was controlled to be comparable to commercial CMC, which could be used in a wide range of applications. Moreover, the findings of the machine learning models highlight the importance of NaOH and H_2_O as the primary factors in the optimization of the pulping process, while AQ and KOH contribute less to the overall process efficiency. The models provided predictions for optimizing the okara pulping process and served as tools for chemical adjustments to achieve the desired yield and quality of extracted cellulose. This research not only highlights a sustainable method for utilizing agricultural waste, but also provides a framework for enhancing process control.

## Figures and Tables

**Table 1:** NaOH, KOH, AQ, and H_2_O variable ranges.

Layer	Variable	Range (%)
Input	NaOH: base chemical	0–50
	KOH: additional chemical	0–50
	AQ: catalyst	0–5
	H_2_O: intermediate	60–100
Output	Yield	20–99

**Table 2:** Key parameters for the random forest regression model.

Parameter	Description
n_estimators	Number of decision trees in the forest. Set to 100 to balance accuracy and computational efficiency.
max_depth	Maximum depth of each tree. Limited to 8 to prevent overfitting.
min_samples_split	Minimum number of samples required to split an internal node. Set to 5 to ensure robustness against noise.
min_samples_leaf	Minimum number of samples required to be at a leaf node. Set to 2 to avoid overly specific splits.
max_features	Number of features to consider when looking for the best split. Set to “auto” to utilize all available features for optimal split decisions.
bootstrap	Whether bootstrap samples are used when building trees. Enabled to increase tree diversity.
criterion	Function to measure the quality of a split. Used “mse” (mean squared error), suitable for regression tasks.

**Table 3:** Selected okara pulping conditions and extracted cellulose yield. NaOH is sodium hydroxide. KOH is potassium hydroxide. AQ is anthraquinone.

Entry	NaOH (%)	KOH (%)	AQ (%)	H_2_O (%)	Temp. (°C)	Yield (%)
1	50	0	-	50	85	26.5
2	40	10	-	50	85	24.0
3	30	10	0.1	59.9	85	24.0
4	30	0	0.1	69.9	85	23.0
5	20	10	0.1	69.9	85	24.5
6	20	0	0.1	79.9	85	26.5
7	10	40	-	50	85	31.0
8	0	50	-	50	85	34.5

**Table 4:** Okara CMC formation conditions with the different ratios between okara pulp and chloroacetic acid. DS is degree of substitution of carboxymethyl cellulose.

Entry	Okara Pulp: Chloroacetic Acid (Ratio)	Okara Pulp: IPA (Ratio)	Yield (wt%)	DS
A	1:1.20	1:20	23.85	0.266
B	1:1.25	1:20	23.64	0.281
C	1:1.50	1:20	23.03	0.348
D	Commercial CMC			0.324

## Data Availability

Data are contained within the article.

## Supplementary Materials

The following supporting information can be downloaded at: https://www.mdpi.com/article/10.3390/polym17060777/s1, Table S1. Case study: process variables.
