# Supplementary material for: Extraction and Conversion of Carboxymethyl Cellulose from Okara Soybean Residue via Soda AQ Pulping: Integration of Predictive Models and Process Control
Source: Polymers (Basel). 2025 Mar 14;17(6):777. doi: 10.3390/polym17060777 (PMC11946626; doi:10.3390/polym17060777)
Supplement: Supplementary file 1 [file polymers-17-00777-s001.zip › polymers-3500782-supplementary.pdf]

Supporting information

Extraction and Conversion of Carboxymethyl Cellulose from Okara Soybean Residue via Soda AQ Pulping: Integration of Predictive Models and Process Control

Preeyanuch Srichola [1,2,*], Titinunt Kitrungrotsakul [3], Kuntawit Witthayolankowit [4], Chaiyaporn Sampoompuang [2], Keowpetch Lobyaem [2], Prapakorn Khamphakun [2] and Rawiwan Tumthong [2]

1 Cellulose for Future Materials and Technologies Special Research Unit, Department of Biotechnology, Faculty of Agro-Industry, Kasetsart University, Chatuchak, Bangkok 10900, Thailand
2 Kasetsart Agricultural and Agro-Industrial Product Improvement Institute, Kasetsart University, Chatuchak, Bangkok 10900, Thailand
3 Research Center for Space Computing System, Zhejiang Lab, Hangzhou 311111, China
4 Department of Chemistry, Faculty of Science, Kasetsart University, Chatuchak, Bangkok 10900, Thailand
* Correspondence: aappua@ku.ac.th

Table S1. Case study: process variables.

NaOH (%)	KOH (%)	AQ (%)	Water (%)	Temp.	Time (hr.)	Yield (%)
20	10	0.1	69.9	85	1	24.5
21	9	0.1	69.9	85	1	23.5
22	8	0.1	69.9	85	1	25
23	7	0.1	69.9	85	1	25
24	6	0.1	69.9	85	1	21
25	5	0.1	69.9	85	1	23
26	4	0.1	69.9	85	1	25
27	3	0.1	69.9	85	1	27
28	2	0.1	69.9	85	1	25
29	1	0.1	69.9	85	1	23
30	0	0.1	69.9	85	1	23
20	0	0.1	79.9	85	1	26.5
21	1	0.1	77.9	85	1	28.5
22	2	0.1	75.9	85	1	26.5
23	3	0.1	73.9	85	1	27
24	4	0.1	71.9	85	1	26

25	5	0.1	69.9	85	1	25
26	6	0.1	67.9	85	1	25
27	7	0.1	65.9	85	1	26.5
28	8	0.1	63.9	85	1	26.5
29	9	0.1	61.9	85	1	25
30	10	0.1	59.9	85	1	24
20	10	0.2	69.8	85	1	26.5
21	9	0.2	69.8	85	1	26.5
22	8	0.2	69.8	85	1	28
23	7	0.2	69.8	85	1	24
24	6	0.2	69.8	85	1	28
25	5	0.2	69.8	85	1	22.5
26	4	0.2	69.8	85	1	24
27	3	0.2	69.8	85	1	22.5
28	2	0.2	69.8	85	1	21.5
29	1	0.2	69.8	85	1	26
30	0	0.2	69.8	85	1	24.5
20	0	0.2	79.8	85	1	24.5
21	1	0.2	77.8	85	1	28
22	2	0.2	75.8	85	1	27
23	3	0.2	73.8	85	1	27
24	4	0.2	71.8	85	1	23.5
25	5	0.2	69.8	85	1	25
26	6	0.2	67.8	85	1	25
27	7	0.2	65.8	85	1	26.5
28	8	0.2	63.8	85	1	25
29	9	0.2	61.8	85	1	24.5
30	10	0.2	59.8	85	1	26.5
20	10	0.3	69.7	85	1	25.5
21	9	0.3	69.7	85	1	28
22	8	0.3	69.7	85	1	27
23	7	0.3	69.7	85	1	27.5

24	6	0.3	69.7	85	1	29.5
25	5	0.3	69.7	85	1	28
26	4	0.3	69.7	85	1	27.5
27	3	0.3	69.7	85	1	26.5
28	2	0.3	69.7	85	1	25.5
29	1	0.3	69.7	85	1	25.5
30	0	0.3	69.7	85	1	25.5
20	0	0.3	79.7	85	1	32.5
21	1	0.3	77.7	85	1	32
22	2	0.3	75.7	85	1	29.5
23	3	0.3	73.7	85	1	26
24	4	0.3	71.7	85	1	28.5
25	5	0.3	69.7	85	1	28.5
26	6	0.3	67.7	85	1	27.5
27	7	0.3	65.7	85	1	28.5
28	8	0.3	63.7	85	1	25
29	9	0.3	61.7	85	1	26.5
30	10	0.3	59.7	85	1	27
20	10	0.4	69.6	85	1	30.5
21	9	0.4	69.6	85	1	29
22	8	0.4	69.6	85	1	29.5
23	7	0.4	69.6	85	1	29.5
24	6	0.4	69.6	85	1	30
25	5	0.4	69.6	85	1	31.5
26	4	0.4	69.6	85	1	31.5
27	3	0.4	69.6	85	1	27.5
28	2	0.4	69.6	85	1	28
29	1	0.4	69.6	85	1	25
30	0	0.4	69.6	85	1	28.5
20	0	0.4	79.6	85	1	35
21	1	0.4	77.6	85	1	30
22	2	0.4	75.6	85	1	32.5

23	3	0.4	73.6	85	1	30
24	4	0.4	71.6	85	1	30.5
25	5	0.4	69.6	85	1	29.5
26	6	0.4	67.6	85	1	29.5
27	7	0.4	65.6	85	1	28
28	8	0.4	63.6	85	1	29
29	9	0.4	61.6	85	1	27.5
30	10	0.4	59.6	85	1	29
20	10	0.5	69.5	85	1	25.5
21	9	0.5	69.5	85	1	26
22	8	0.5	69.5	85	1	26
23	7	0.5	69.5	85	1	28
24	6	0.5	69.5	85	1	28.5
25	5	0.5	69.5	85	1	29.5
26	4	0.5	69.5	85	1	26.5
27	3	0.5	69.5	85	1	26.5
28	2	0.5	69.5	85	1	26.5
29	1	0.5	69.5	85	1	26.5
30	0	0.5	69.5	85	1	29.5
20	0	0.5	79.5	85	1	30
21	1	0.5	77.5	85	1	30
22	2	0.5	75.5	85	1	33
23	3	0.5	73.5	85	1	32.5
24	4	0.5	71.5	85	1	30
25	5	0.5	69.5	85	1	33
26	6	0.5	67.5	85	1	29.5
27	7	0.5	65.5	85	1	30
28	8	0.5	63.5	85	1	29
29	9	0.5	61.5	85	1	28.5
30	10	0.5	59.5	85	1	30
20	10	0.6	69.4	85	1	26.5
21	9	0.6	69.4	85	1	26.5

22	8	0.6	69.4	85	1	33.5
23	7	0.6	69.4	85	1	32
24	6	0.6	69.4	85	1	32.5
25	5	0.6	69.4	85	1	31.5
26	4	0.6	69.4	85	1	30.5
27	3	0.6	69.4	85	1	32
28	2	0.6	69.4	85	1	31.5
29	1	0.6	69.4	85	1	30.5
30	0	0.6	69.4	85	1	30
20	0	0.6	79.4	85	1	36.5
21	1	0.6	77.4	85	1	29.5
22	2	0.6	75.4	85	1	31.5
23	3	0.6	73.4	85	1	32.5
24	4	0.6	71.4	85	1	30.5
25	5	0.6	69.4	85	1	30
26	6	0.6	67.4	85	1	29.5
27	7	0.6	65.4	85	1	30
28	8	0.6	63.4	85	1	29
29	9	0.6	61.4	85	1	29
30	10	0.6	59.4	85	1	32.5
40	10	0	50	85	1	24
40	10	1	49	85	1	23
40	10	2	48	85	1	24.5
40	10	3	47	85	1	22
40	10	4	46	85	1	25
40	10	5	45	85	1	27
50	10	0	40	85	1	23
50	0	1	49	85	1	24
50	0	2	48	85	1	23
50	0	3	47	85	1	21.5
50	0	4	46	85	1	24
50	0	5	45	85	1	24

10	40	0	50	85	1	31
10	40	1	49	85	1	30.5
10	40	2	48	85	1	33
10	40	3	47	85	1	31.5
10	40	4	46	85	1	33.5
10	40	5	45	85	1	34
0	50	0	50	85	1	33
0	50	1	49	85	1	33.5
0	50	2	48	85	1	34.5
0	50	3	47	85	1	33.5
0	50	4	46	85	1	35.5
0	50	5	45	85	1	35.5
0	0	0	100	85	1	87
0	0	0.5	99.5	85	1	98.5
0	0	1	99	85	1	98
0	0	2	98	85	1	97.5
50	0	0	50	85	1	26.5
0	50	0	50	85	1	34.5
50	0	1	49	85	1	25.5
0	50	1	49	85	1	35
